# Prediction of mortality in severe acute malnutrition in hospitalized children by faecal volatile organic compound analysis: proof of concept

**DOI:** 10.1038/s41598-020-75515-6

**Published:** 2020-11-05

**Authors:** Deborah A. van den Brink, Tim de Meij, Daniella Brals, Robert H. J. Bandsma, Johnstone Thitiri, Moses Ngari, Laura Mwalekwa, Nanne K. H. de Boer, Alfian Wicaksono, James A. Covington, Patrick F. van Rheenen, Wieger P. Voskuijl

**Affiliations:** 1 Department of Paediatrics, Centre for Liver, Digestive and Metabolic Diseases, University of Groningen, University Medical Centre Groningen, Groningen, The Netherlands; 2 Amsterdam Centre for Global Child Health, Emma Children’s Hospital, Amsterdam University Medical Centres, Amsterdam, The Netherlands; 3 Department of Global Health, Amsterdam Institute for Global Health and Development, Amsterdam University Medical Centres, Amsterdam, The Netherlands; 4 Department of Paediatric Gastroenterology, Emma, Children’s Hospital, Amsterdam University Medical Centres, Amsterdam, The Netherlands; 5 Department of Gastroenterology and Hepatology, Amsterdam Gastroenterology and Metabolism Research Institute, Amsterdam University Medical Centres, Amsterdam, The Netherlands; 6 Division of Gastroenterology, Hepatology and Nutrition and Translational Medicine Program, Hospital for Sick Children, Toronto, Canada; 7 The Childhood Acute Illness & Nutrition Network (CHAIN), Nairobi, Kenya; 8 KEMRI/Welcome Trust Research Programme, Kilifi, Kenya; 9 Department of Paediatrics, College of Medicine, University of Malawi, Blantyre, Malawi; 10 Department of Biomedical Sciences, College of Medicine, University of Malawi, Blantyre, Malawi; 11 School of Engineering, University of Warwick, Coventry, UK

**Keywords:** Computational science, Malnutrition, Biomarkers, Paediatric research, Translational research

## Abstract

Children with severe acute malnutrition (SAM) display immature, altered gut microbiota and have a high mortality risk. Faecal volatile organic compounds (VOCs) reflect the microbiota composition and may provide insight into metabolic dysfunction that occurs in SAM. Here we determine whether analysis of faecal VOCs could identify children with SAM with increased risk of mortality. VOC profiles from children who died within six days following admission were compared to those who were discharged alive using machine learning algorithms. VOC profiles of children who died could be separated from those who were discharged with fair accuracy (AUC) = 0.71; 95% CI 0.59–0.87; *P* = 0.004). We present the first study showing differences in faecal VOC profiles between children with SAM who survived and those who died. VOC analysis holds potential to help discover metabolic pathways within the intestinal microbiome with causal association with mortality and target treatments in children with SAM.

**Trial Registration:** The F75 study is registered at clinicaltrials.gov/ct2/show/NCT02246296.

## Introduction

While there has been a substantial improvement in the under-five mortality rate over the past decades, still 16,000 children die worldwide daily, of which under-nutrition is considered a key factor in almost 50%^1^. Sub-Saharan Africa is hit the hardest, where 1 in every 12 children will die before their fifth birthday^1^. Undernourished children can be classified as either being moderately malnourished (moderate acute malnutrition or MAM), or severely malnourished (severe acute malnutrition or SAM). Complicated SAM (with medical complications such as systemic or respiratory infection or profound diarrhoea) requires in-patient treatment^2^. Even under strict adherence to treatment guidelines, case fatality rate for patients with complicated SAM in African hospitals remains high (> 20%)^3,4^. So, there is an urgent need to improved understanding of the pathophysiology in this vulnerable group of children as well as better identification of children with SAM with the highest mortality. Several risk factors have been associated with this persistent high mortality, including HIV, very low anthropometry, oedema, and gastro-intestinal dysfunction leading to diarrhoea, present in roughly half of SAM patients. However clinical models, for high-accuracy prediction of mortality and understanding its mechanisms in SAM, are not well validated nor established so far^5,6^. Increasing evidence suggests that the gut microbiota plays a crucial etiological role in gastrointestinal dysfunction^7–12^. Studies in Bangladeshi and Ugandan children revealed that malnourished children had an ‘immature’ microbiota, characterized by decreased microbial diversity^9,10^. Identification of a microbial ‘signature’ associated with increased risk for mortality could hypothetically select high-risk SAM patients, increase our understanding of pathophysiology and open avenues towards development of targeted therapeutic interventions aimed at reducing mortality rates.

Volatile organic compounds (VOCs) are carbon-based molecules originating from metabolic processes in the human body and reflect microbiota composition, metabolic function, and interaction with the host^13^. Faecal VOC analysis has been shown to have potential as a diagnostic biomarker (i.e. monitor gut changes non-invasively) particularly for diseases in which microbiota alterations are considered to play an etiological role, including (paediatric) inflammatory bowel disease, necrotizing enterocolitis, colorectal cancer, and sepsis^14–18^. Faecal VOCs are produced in the gastrointestinal tract mainly by residing microbes, fermentation of non-starch polysaccharides, as well as the hosts response to changes in gut bacterial compositions and health. In order to establish this method, we explored the potential of faecal VOCs as a non-invasive measure for predicting mortality in malnourished children. We hypothesized that faecal VOCs from survivors of SAM differ from non-survivors.

## Results

Characteristics of 57 patients, including by survival outcome are presented in Table 1. The mean age among children who were discharged (*n* = 38) was 25.9 months and children in this group were discharged from the hospital after on average 6.9 days. Children who died (*n* = 19) were significantly younger than children discharged, with a mean age of 16.8 months (*P* = 0.04), had a lower MUAC at admission (*P* = 0.03), and were more likely to have early warning signs upon admission (*P* = 0.006), as compared to the discharged children.

**Table 1:** Characteristics of study participants upon admission and by outcome (discharged vs. died).

	SAM	Discharged	Died	*P*
	*N* = 57	*N* = 38	*N* = 19	
**Study site [n (%)]**				
Coast Provincial General Hospital	25 (43.9)	15 (39.5)	10 (52.6)	
Kilifi County Hospital	5 (8.8)	4 (10.5)	1 (5.3)	
Queen Elizabeth Central Hospital	27 (47.4)	19 (50.0)	8 (42.1)	0.59
Age [mean (SD)], months	22.9 (15.7)	25.9 (16.1)	16.8 (13.3)	0.04
Male [n (%)]	35 (61.4)	23 (60.5)	12 (63.2)	0.85
Fully breastfed [n (%)]	23 (40.4)	14 (36.8)	9 (47.4)	0.45
**Anthropometrics [mean (SD)]**				
MUAC cm	11.2 (1.6)	11.5 (1.6)	10.5 (1.5)	0.03
Height-for-age z-score	55; − 3.1 (1.7)	38; − 3.2 (1.5)	17; − 3.1 (2.3)	0.9
Weight-for-age z-score	− 4.0 (1.4)	− 3.8 (1.3)	− 4.3 (1.6)	0.17
Weight-for-height z-score	53; − 3.3 (1.4)	37; − 3.1 (1.4)	16; − 3.7 (1.4)	0.19
Oedema [n (%)]	22 (38.6)	17 (44.7)	5 (26.3)	0.18
Vomiting [n (%)]	14 (24.6)	7 (18.4)	7 (36.8)	0.13
Diarrhoea [n (%)]	23 (40.4)	12 (31.6)	11 (57.9)	0.06
**HIV test result [n (%)]**				
Negative	38 (66.7)	28 (73.7)	10 (52.6)	
Positive	14 (24.6)	7 (18.4)	7 (36.8)	
Refusal or died before HIV testing	5 (8.8)	3 (7.9)	2 (10.5)	0.26
Tuberculosis [n (%)]	1 (1.8)	1 (2.6)	0 (0.0)	0.48
Fever [T > 38 °C; n (%)]	19 (33.3)	9 (23.7)	10 (52.6)	0.03
Severe pneumonia [n (%)]	17 (29.8)	10 (26.3)	7 (36.8)	0.42
Any danger signs at admission [n (%)]*	8 (14.0)	2 (5.3)	6 (31.6)	0.006

*MUAC* mid upper arm circumference, *HIV* human immunodeficiency virus 1.

*World Health Organisation (WHO) danger signs suggestive of systemic illness or clinical deterioration (respiratory distress, profuse diarrhoea, hypoglycaemia, tachycardia, etc).

Healthy control children were older (*P* < 0.001) and had a higher MUAC (*P* < 0.001), as compared to SAM patients (see Table 2).

**Table 2:** Characteristics of study participants with SAM and healthy control children.

	Healthy controls	SAM	*P*
	N = 7	*N* = 57	
Age [months; mean (SD)]	52.9 (22.1)	22.9 (15.7)	< 0.001
Male [*n* (%)]	4 (57.1)	35 (61.4)	0.83
MUAC [mean (SD)]	15.1 (2.0)	11.2 (1.6)	< 0.001

*MUAC* mid upper arm circumference.

**Figure 1:**
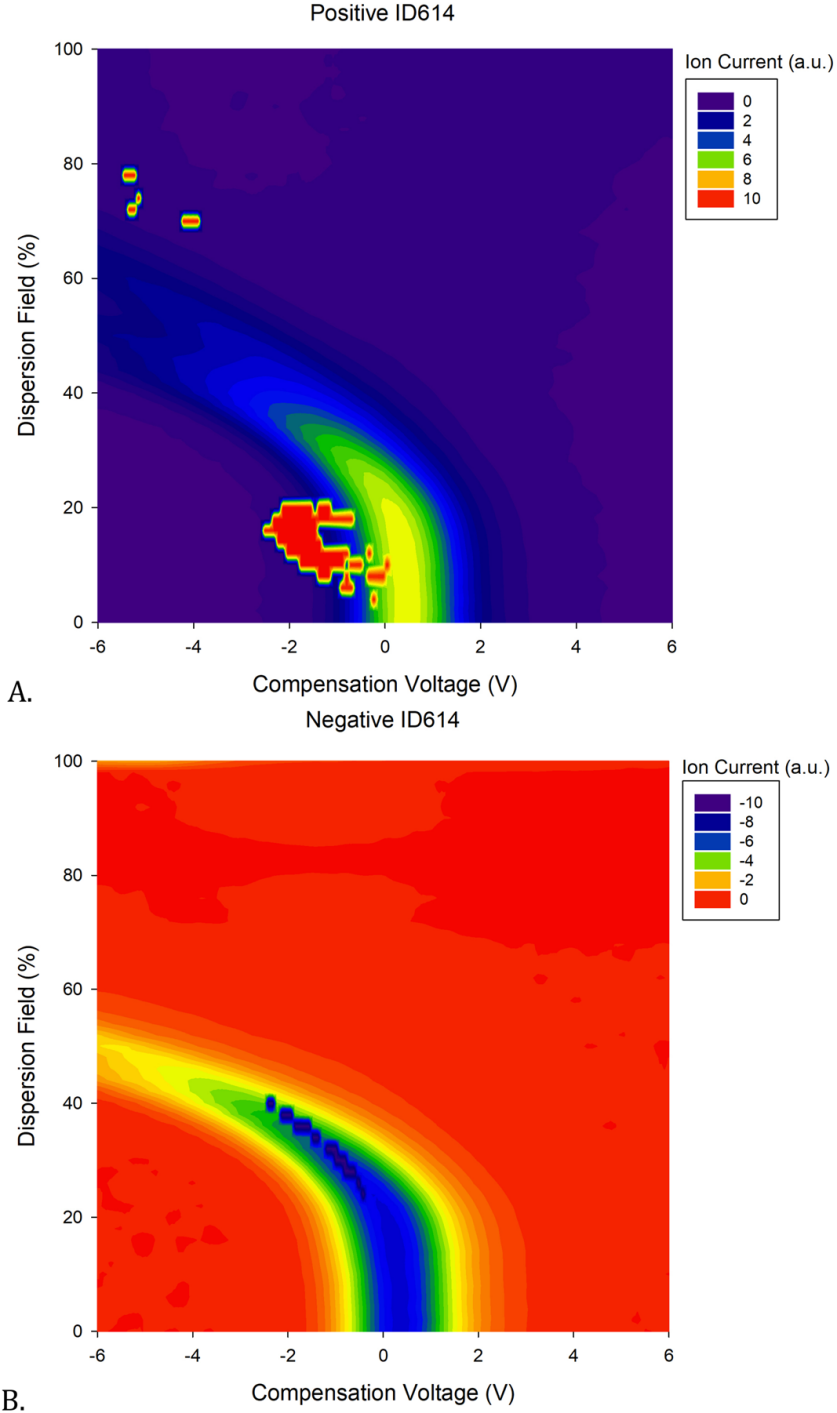
Feature map illustrating locations on the FAIMS output from children who died within 6 days of admission. (**A**) Positive feature locations (**B**) Negative feature locations.

Analysis was conducted on 100 features, 50 features, and 20 features. A feature map illustrating locations on the FAIMS output of VOC profiles from children who died within 6 days following admission compared to those who were discharged alive can be seen in Fig. 1.

**Figure 2:**
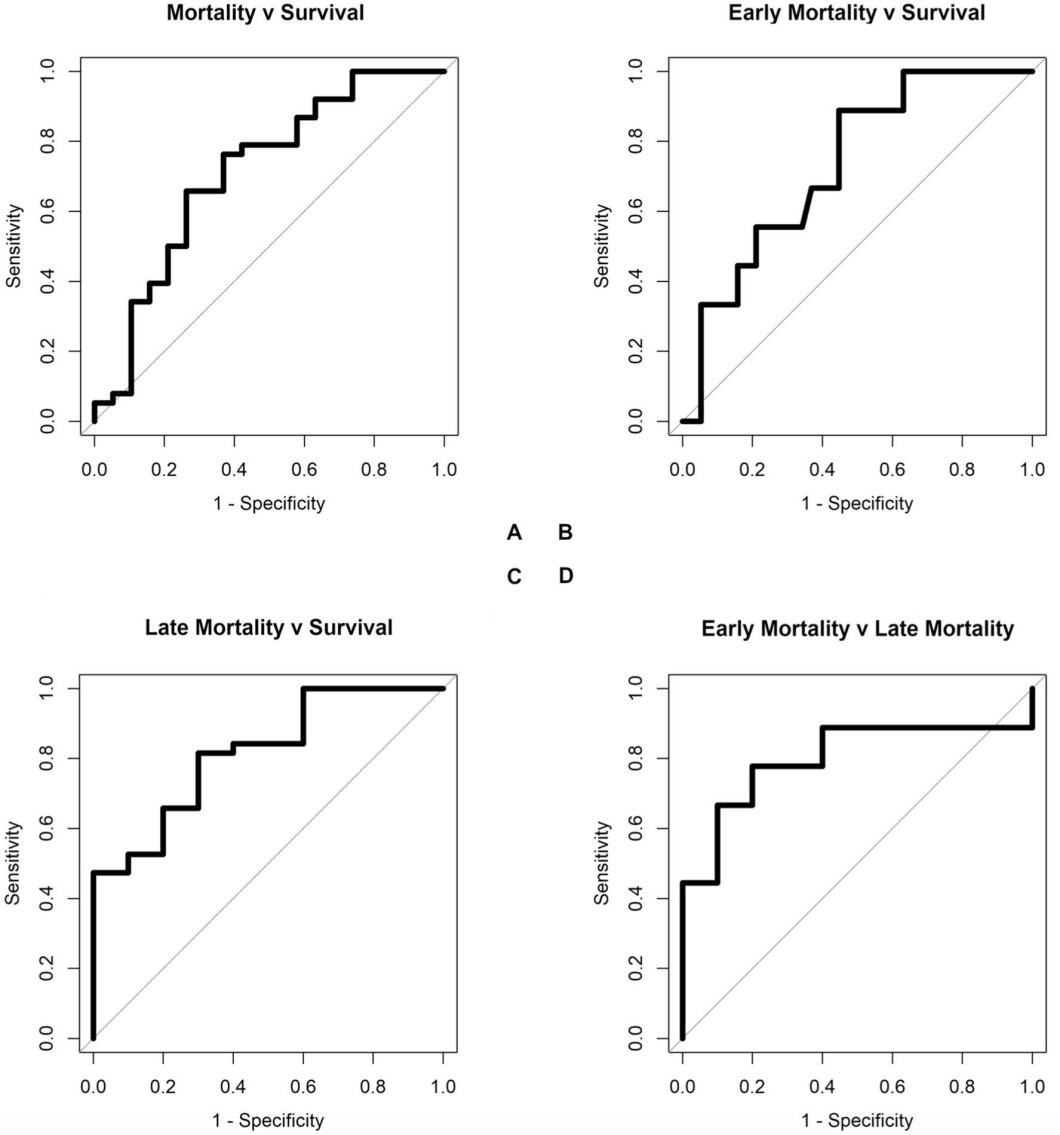
(**A**) Mortality v survival. Support vector machine (100 features). (**B**) Early mortality v survival. Random forest (100 features). (**C**) Late mortality v survival. Sparse logistic regression (50 features). (**D**) Early mortality v late mortality. Support vector machine (50 features).

Four different classifiers were run for each comparison, and the best performing machine learning classifications are shown in Table 3 and Fig. 2. The results of all applied classifiers for each comparison can be found in Table S1 in the Supplementary Data. VOC profiles of children dying on day 4, 5 or 6 of admission (‘late’ mortality) could be separated from the VOC profiles of children who were discharged with high accuracy [area under the receiver operating characteristic curve (AUC) 0.82; 95% CI 0.67–0.96; *P* < 0.001], whereas VOC profiles of children dying within the first 3 days of admission could be separated from the VOC profiles children who were discharged with fair accuracy (AUC = 0.73; 95% CI 0.57–0.9, *P* = 0.02). We were also able to separate early mortality from late mortality with high accuracy (AUC = 0.8; 95% CI 0.57–1; *P* = 0.001).

**Table 3:** Machine learning classification results.

	Best performing algorithm	Features (number)	AUC (95% CI)	P	Sensitivity (95% CI)	Specificity (95% CI)	PPV	NPV
**Mortality classifications**								
Mortality vs. survival	Support vector machine	100	0.71 (0.56–0.87)	0.004	0.76 (0.6–0.89)	0.63 (0.38–0.84)	0.81	0.57
Early mortality vs. survival	Random forest	100	0.73 (0.57–0.9)	0.02	0.89 (0.52–1)	0.55 (0.38–0.71)	0.32	0.95
Late mortality vs. survival	Sparse logistic regression	50	0.82 (0.67–0.96)	< 0.001	0.82 (0.66–0.92)	0.7 (0.35–0.93)	0.91	0.5
Early vs. late mortality	Support vector machine	50	0.8 (0.57–1)	0.001	0.78 (0.4–0.97)	0.8 (0.44–0.97)	0.78	0.8
**Morbidity classifications**								
SAM vs. healthy controls	Sparse logistic regression	100	0.99 (0.98–1)	< 0.001	0.96 (0.88–1)	1 (0.59–1)	1	0.78
WAZ ≤ − 3 vs. WAZ > − 3	Sparse logistic regression	100	0.7 (0.54–0.86)	0.02	0.73 (0.39–0.94)	0.7 (0.54–0.82)	0.36	0.91
Oedema vs. no oedema	Sparse logistic regression	20	0.71 (0.56–0.87)	0.003	0.77 (0.55–0.92)	0.66 (0.48–0.81)	0.59	0.82
Diarrhoea vs. no diarrhoea	Support vector machine	100	0.66 (0.51–0.81)	0.02	0.45 (0.24–0.68)	0.89 (0.73–0.97)	0.71	0.72
Pneumonia vs. no pneumonia	Gaussian process	100	0.63 (0.47–0.75)	0.06	0.59 (0.33–0.82)	0.73 (0.56–0.85)	0.48	0.81
HIV + vs. HIV −	Support vector machine	100	0.73 (0.58–0.87)	0.01	0.93 (0.66–1)	0.53 (0.36–0.69)	0.42	0.95
Age > 2 yrs. vs. age ≤ 2 yrs	Random forest	20	0.79 (0.66–0.92)	< 0.001	0.75 (0.51–0.91)	0.76 (0.59–0.88)	0.63	0.85

*AUC* area under the receiver operating characteristic curve, *WAZ* weight-for-age z-score, *HIV* human immunodeficiency virus 1, *yrs.* years.

Definitions: early mortality = mortality within 3 days of admission; late mortality = mortality on day 4, 5, or 6 of admission.

Healthy controls (siblings) could be separated from children with complicated SAM with very high accuracy (AUC = 0.99; 95% CI 0.98–1; *P* < 0.001). In addition, the classifiers were also able to separate the following VOC profiles: WAZ ≤ − 3 form WAZ > − 3 (AUC = 0.7; 95% CI 0.54–86; *P* = 0.02), oedema from no oedema (AUC = 0.71; 95% CI 0.56–0.87; *P* = 0.003), diarrhoea from no diarrhoea (AUC = 0.66; 95% CI 0.51–0.81; *P* = 0.02), HIV positive from HIV negative (AUC = 0.73; 95% CI 0.58–0.87; *P* = 0.01), and age ≤ 2 years months from age > 2 years (AUC = 0.79; 95% CI 0.66–0.89; *P* < 0.001). Only the VOC profiles of children with pneumonia could not be separated from the VOC profiles of children without pneumonia (AUC = 0.63; 95% CI 0.47–0.75; *P* = 0.06).

## Discussion

This is the first study showing differences in faecal VOC profiles between children with SAM who survived and those who died, and this likely reflects microbiota composition differences between these 2 groups. Fecal VOCs of children who died from SAM could be separated from children who were discharged with fair accuracy. Discriminative accuracy increased even further to high accuracy when taking only VOC profiles of late mortality subjects into account.

Current evidence supports the increasing notion that children with SAM have a specific, altered metabolic and microbial signature compared to non-malnourished children^19–25^. By non-invasive VOC analysis we were able to run algorithms that predicted mortality with a fair AUCs as well as a high degree of sensitivity and specificity. Noteworthy was the finding that the difference in VOC-profiles between children who died and those surviving to discharge became more pronounced when the longer interval between admission and dying was chosen (6 versus 3 days). This observation warrants further investigation as we would assume that volatiles associated with an increased risk for dying would be higher among early mortality patients. Another explanation could be that early mortality is more impacted by the acute illness and later mortality more to microbiome-related effects.

We also grouped VOC profiles according to other important clinical characteristics (and known contributors to mortality) in order to ascertain that observed differences between the mortality and survival group could not be solely attributed to one of these factors (i.e. age, oedema, HIV, diarrhoea, and low weight-for-height). The SAM oedematous phenotype (kwashiorkor) is known to have a different microbiota profile compared with non-oedematous malnourished children (marasmus)^7,8,10,19^ and we were able to show this with these preliminary data. Also, HIV positive children could be discriminated from HIV negative children confirming HIV disrupts intestinal immunity, which can lead to chronic inflammation^26^, and microbial dysbiosis^27^. Antibiotics are another factor that can influence the microbiome^28–31^, and while samples were collected at admission, many children might have already been given antibiotics prior to referral to our study sites. Future studies would need to look at the effect of both antibiotic use and the use of different Microbiota-Directed Complementary Foods^11,12^ on VOCs, both qualitative as quantitative.

Growth and health of children is functionally associated to microbial changes (including maturation)^7^. Malnourished children have an immature and altered microbiome^10,19^, as well as an increased likelihood of metabolic dysfunction^32^. VOCs are not merely produced by gut microbiota alone, but may at least partly result from the intestinal mucosal inflammatory process and metabolic alterations associated with SAM. Further studies are needed to address the specific VOCs leading to observed differences next to unravelling the (micro-biotic) origin of these volatiles. Identification of specific VOCs associated with mortality may allow for enhanced understanding of pathophysiological processes underlying different pathways in children with SAM as well as development of tailor-made sensors to be used as handheld VOC analyser in clinical practice (as an early prediction tool).

Strength of this study is that samples were used from 3 sites across 2 countries in sub-Saharan Africa, allowing to capture VOC profiles of different African SAM populations^33^. Our patients had an extensive and detailed prospective collection of clinical data and our machine learning algorithms have been validated in other paediatric populations^13,14,16,34^. Children who died and those who survived had mostly similar baseline characteristics (including the use of antibiotics) making the (interpretation of) VOC differences even stronger. Finally, collection, storage and transport of the samples were performed strictly according standardized protocols, while faecal VOC analysis was performed using optimal sampling conditions according to reference values as described previously^35^.

Our study has limitations as well. First, the number of included patients was a relatively small, biased set from a larger study, and our findings need to be validated in a larger external cohort, preferably including children with SAM from different geographical areas. Another limitation is that FAIMS technology allows for rapid analysis of the complete spectrum of volatile molecules, but does not allow for identification of individual compounds contributing to the observed differences in VOC profiles. We did however cluster patients according to (clinical) characteristics associated with increased mortality in complicated SAM in an attempt to bring forward some theories as to what signals or factors are underlying these signals. The healthy controls were much older than the children with SAM, and the children who died were younger than those discharged alive. Finally, the lack of microbiome data is also limiting interpretation of our results. We also acknowledge the limitation of our small control group of 7 children. This is a small group when using machine learning but we believe that inclusion of this small control group was still important for the study.

This study brings forth an exciting discovery that VOC analysis is able to detect altered metabolic signals from the microbiota that are linked to mortality in SAM. With future studies that are able to separate the individual components of these altered signals we hope to identify specific compounds and metabolites that are linked to mortality in SAM. This would improve our understanding of underlying, pathophysiological pathways to mortality in children with SAM. Once potential mechanisms are established this could lead to better targeted treatment and potentially identify high-risk patients early on admission; both aiming at reducing the current unacceptable high mortality rates. Conversely, identification of low-risk children with SAM could lower the overall burden of clinical care, might prevent the need for broad-spectrum antibiotics and facilitate earlier discharge. Future larger scale research on the risk stratifying purpose of VOCs is needed to validate these results both in African as well as Asian populations with different microbial profiles^36^.

## Methods

### Study population

This was a case–control study, matched by site and sex, using faecal samples of 57 children included in a multicentre randomized, double blinded intervention study (F75 study, ClinicalTrials.gov; no. NCT02246296). Children were enrolled in 3 centres: Queen Elizabeth Hospital, in Malawi; Kilifi County Hospital in Kenya, and Coast Provincial General Hospital in Kenya. The “F75 study” included 843 patients and evaluated whether modified F75 formula would decrease the time to clinical stabilization compared to the standard F75 nutrition rehabilitation formula^33^.

Inclusion criteria for the original F75 study were as follows: children aged 6 months to 13 years, classified as complicated SAM with either medical complications or failing an appetite test, who were admitted to the malnutrition ward^33^. SAM was defined as a mid-upper arm circumference (MUAC) score < 11.5 cm, or a weight-for-height z-score WHZ (WHZ) <  − 3, or/and bilateral oedema according to WHO guidelines^2,37^. All children were placed on a F75 formula, a standardized WHO refeeding formula which was produced by Nutriset (Nutriset, Malaunay, France) which was given every 3 h. There were two different formulas used where protein was consistent at 5.3%^33^. F75 formula has 31.5% lipids and 63.2% carbohydrates, whereas the modified F75 contained 51.7% lipids and 43% carbohydrates^33^. Children were randomized to both milk formulas for the study. Later on during admission children were placed on standardized Ready to Use Therapeutic Foods (RUTF), also given every 3 h. Informed consent was obtained from parents prior to enrolment in the study. Both HIV-positive and HIV-negative children were included in the study. Ethical approval was obtained from the College of Medicine Research Ethics Committee of the University of Malawi, the KEMRI Ethical Review Committee in Kenya, the Oxford Tropical Research Ethics Committee, and the Hospital for Sick Children, Toronto. This study was carried out in accordance to the regulations of each respective country and ethical committee.

The 57 faecal samples analysed in the present study were selected in the following manner: first, 72 children from the original F75 trial, aged between 6 months and 5 years, that had died within 6 days of admission were randomly selected, and then matched by site and sex, with children who were discharged from the hospital.

For the measurement, we needed a faecal sample size of at least 0.4 g which limited our original matched selection, and resulted in 19 faecal samples of children who died within 6 days after admission (cases) and 38 faecal samples of children who were discharged alive.

Faecal samples of 7 healthy siblings of SAM patients recruited at Queen Elizabeth Hospital served as a healthy control group since it is known that the microbiota of healthy children do differ significantly from children with SAM. Eligibility to serve as a healthy control was as follows: sibling of a F75 study patient, between 6 months and 6 years of age, WHZ >  − 2, MUAC > 12.5 cm, no oedema, no hospital admission in the last year, no diarrhoea in the past month, and no fever in the past month. Since this was an initial proof of principle study, no formal sample size calculation was performed.

### Clinical data and biological sample collection

At admission to hospital, comprehensive clinical and anthropometric data were collected and recorded including appetite and dietary data, anthropometric data, degree of oedema, medical complications, and comorbidities, and prior antibiotic prescription. For a complete list of variables see Online Online Appendix 2: Table S2. Stool samples were collected on admission day.

### VOC analysis by field asymmetric ion mobility spectrometry (FAIMS) technology

VOC analysis was undertaken by Ion Mobility Spectrometry, specifically using a FAIMS technique. Here a commercial system was used, which is a portable, self-contained unit (Lonestar with ATLAS sampling system, Owlstone Ltd., UK). FAIMS is able to separate complex mixtures of chemicals through a combination of ionisation followed by measuring the difference in ion mobility in high-electric fields^38^. We have used this technique over more traditional analytical approaches at it has high sensitivity, rapid/simple sample throughput (e.g. uses air as the carrier) and lower sampling/unit cost. The ionisation process is undertaken through the exposure of the gaseous species to a radioactive source (Ni-63 in our case). The resultant ions are then pushed between two plates onto which an asymmetric electric field is applied, comprising of a short high potential being applied in one direction and longer lower potential applied in the opposite direction (but with the period × applied potential being equal). This results in the ions moving between the plates (in a zig-zag pattern) and are detected as they exit the plates. These ions can be attracted, repelled or not affected by the difference in electric field depending on its properties. Any ion that collides with a plate loses its charge and is not detected. To counteract any movement of the ions, a compensation voltage is applied (from + 6 V to − 6 V in 512 steps). This scanning process allows ions of different mobilities to be detected. Furthermore, the magnitude of the electric field was also scanned from 0 to 100% in 51 steps (as ion movement in non-linear with electric field) to further increase the information content. As both positive and negative ions are measured, the total number of data points per sample is 52,224. Each sample was tested 3 times, with the second sample used. From previous studies, we have found that this second sample provides the most useful discriminatory information.

### Procedures

Faecal samples were collected at admission to the hospital, homogenised, aliquoted into cryovials, and stored at – 80 °C within 30 min after collection. They were transported on dry ice by a certified courier from Malawi and Kenya to The Netherlands and thawed prior to analysis with the Lonestar. VOC Analyses were performed in December 2017. Faecal samples were defrosted on ice 1–2 h prior to the VOC analysis. Approximately 0.40 g of faeces was weighed out with a 15% error margin. 10 ml of sterilised tap water was mixed in with the sample in a sterilized glass jar. The flow rate was consistent across the samples, with temperatures being set at 35 °C for the sample, 70 °C for the transfer unit, and 100 °C for the inlet filter temperatures. This protocol was consistent with methodology applied in previous studies and based upon outcome of a study on optimized sampling conditions in faecal VOC analyses using FAIMS^14,16,35,38^.

### Statistical analysis

Our primary outcomes were: SAM versus healthy controls (validation); mortality within 6 days versus discharge (survival); mortality within 3 days (i.e. early mortality) versus discharge; mortality before within 6 days (on day 4, 5, or 6 i.e. late mortality) versus discharge; early mortality versus late mortality. As mortality in children with SAM is multi-factorial, we included secondary outcomes which are known risk factors for mortality in this population. Secondary outcomes were: weight-for-age z-score (WAZ) ≤  − 3 standard deviation (SD) versus WAZ >  − 3 SD; oedema versus no oedema; diarrhoea versus no diarrhoea; pneumonia versus no pneumonia; HIV positive versus HIV negative; age ≤ 2 years versus age > 2 years. Baseline characteristics were compared using T-tests.

Though the FAIMS technique is highly sensitive, it is unable to identify specific chemicals and thus a pattern recognition technique was applied. To this end, we have developed a data analysis pipeline to undertake this task, which has been used on a number of previous studies. The detailed steps can be found in previous reports^14,16,18,38^. In brief, first both the positive and negative ion data are combined together to create a single 2D array for each sample. We then applied a threshold to remove the background/areas that contain no information to reduce the computational overhead of the following steps. Then a tenfold cross validation approach is applied. Here the data is split into a 90% training set and a 10% test set. To the training set, a rank-sum test is applied to each data point to identify the top 100 data points/features that contain the most discriminatory information. These features are then used to train four different classifiers (specifically: Random Forest, Gaussian Process Classifier, Support Vector Machine, and Sparse Logistic Regression. This is part of our standard pipeline), which are then applied to the test set. This process is repeated 10 times until all the samples are classified as test samples and as the feature selection is within the fold, it reduces issues associated with over-fitting of data. The resultant data is then used to calculate statistical parameters, such as sensitivity and specificity.

From there, several machine learning algorithms using only the VOC data were used to determine whether the sub-groups could be separated based on faecal VOC profiles.

## Supplementary information

Supplementary Legends.

Supplementary Information 1.

Supplementary Information 2.

## Data Availability

All machine learning results are available in the Supplementary Tables. The raw VOC data is also available in the supplementary data (Supplement 3).

## References

[1] UNICEF (2015). Levels and Trends in Child Mortality.

[2] WHO (2013). Guideline: Updates on the Management of Severe Acute Malnutrition in Infants and Children.

[3] Heikens GT (2008). Case management of HIV-infected severely malnourished children: Challenges in the area of highest prevalence. Lancet (London, England).

[4] Brewster DR (2011). Inpatient management of severe malnutrition: Time for a change in protocol and practice. Ann. Trop. Paediatr..

[5] Kerac M (2014). Follow-up of post-discharge growth and mortality after treatment for severe acute malnutrition (FuSAM study): A prospective cohort study. PLoS ONE.

[6] Probert CSJ (2009). Volatile organic compounds as diagnostic biomarkers in gastrointestinal and liver diseases. J. Gastrointest. Liver Dis..

[7] Blanton LV, Barratt MJ, Charbonneau MR, Ahmed T, Gordon JI (2016). Childhood undernutrition, the gut microbiota, and microbiota-directed therapeutics. Science.

[8] Smith MI (2013). Gut microbiomes of Malawian twin pairs discordant for kwashiorkor. Science.

[9] Kristensen KHS (2016). Gut microbiota in children hospitalized with oedematous and non-oedematous severe acute malnutrition in Uganda. PLoS Negl. Trop. Dis..

[10] Blanton LV (2016). Gut bacteria that prevent growth impairments transmitted by microbiota from malnourished children. Science.

[11] Raman AS (2019). A sparse covarying unit that describes healthy and impaired human gut microbiota development. Science.

[12] Gehrig JL (2019). Effects of microbiota-directed foods in gnotobiotic animals and undernourished children. Science.

[13] Buijck M (2016). Sniffing out paediatric gastro-intestinal diseases: The potential of volatile organic compounds as biomarkers for disease. J. Pediatr. Gastroenterol. Nutr..

[14] van Gaal N (2017). Faecal volatile organic compounds analysis using field asymmetric ion mobility spectrometry: Non-invasive diagnostics in paediatric inflammatory bowel disease. J. Breath Res..

[15] De Meij TGJ, Boer NKH, Benninga MA, Bodegraven AA, Schee MP (2014). P-008: Fecal gas analysis by electronic nose: A novel, non-invasive technique for assessment of active and quiescent pediatric inflammatory bowel disease. J. Crohn’s Colitis.

[16] Berkhout DJC (2017). Detection of sepsis in preterm infants by fecal volatile organic compounds analysis: A proof of principle study. J. Pediatr. Gastroenterol. Nutr..

[17] de Meij TGJ (2015). Early detection of necrotizing enterocolitis by fecal volatile organic compounds analysis. J. Pediatr..

[18] de Meij TG (2014). Electronic nose can discriminate colorectal carcinoma and advanced adenomas by fecal volatile biomarker analysis: Proof of principle study. Int. J. Cancer.

[19] Subramanian S (2014). Persistent gut microbiota immaturity in malnourished Bangladeshi children. Nature.

[20] Bartz S (2014). Severe acute malnutrition in childhood: Hormonal and metabolic status at presentation, response to treatment, and predictors of mortality. J. Clin. Endocrinol. Metab..

[21] Owino V (2016). Environmental enteric dysfunction and growth failure/stunting in global child health. Pediatrics.

[22] Tickell KD, Denno DM (2016). Inpatient management of children with severe acute malnutrition: A review of WHO guidelines. Bull. World Health Organ..

[23] Tilg H, Moschen AR (2013). Malnutrition and microbiota—A new relationship?. Nat. Rev. Gastroenterol. Hepatol..

[24] Murray E, Manary M (2015). Possible role of the microbiome in the development of acute malnutrition and implications for food-based strategies to prevent and treat acute malnutrition. Food Nutr. Bull..

[25] Freemark M (2015). Metabolomics in nutrition research: Biomarkers predicting mortality in children with severe acute malnutrition. Food Nutr. Bull..

[26] Zilberman-Schapira G (2016). The gut microbiome in human immunodeficiency virus infection. BMC Med..

[27] Bandera A, De Benedetto I, Bozzi G, Gori A (2018). Altered gut microbiome composition in HIV infection: Causes, effects and potential intervention. Curr. Opin. HIV AIDS.

[28] Vemuri R (2018). Gut microbial changes, interactions, and their implications on human lifecycle: An ageing perspective. Biomed. Res. Int..

[29] Willing BP, Russell SL, Finlay BB (2011). Shifting the balance: Antibiotic effects on host-microbiota mutualism. Nat. Rev. Microbiol..

[30] Cox LM (2014). Altering the intestinal microbiota during a critical developmental window has lasting metabolic consequences. Cell.

[31] Cully M (2019). Antibiotics alter the gut microbiome and host health. Nat. Res..

[32] Attia S (2016). Mortality in children with complicated severe acute malnutrition is related to intestinal and systemic inflammation: An observational cohort study. Am. J. Clin. Nutr..

[33] Bandsma RHJ (2019). A reduced-carbohydrate and lactose-free formulation for stabilization among hospitalized children with severe acute malnutrition: A double-blind, randomized controlled trial. PLoS Med..

[34] de Meij TGJ (2016). Characterization of microbiota in children with chronic functional constipation. PLoS ONE.

[35] Bosch S (2018). Optimized sampling conditions for fecal volatile organic compound analysis by means of field asymmetric ion mobility spectrometry. Anal. Chem..

[36] Pop M (2014). Diarrhea in young children from low-income countries leads to large-scale alterations in intestinal microbiota composition. Genome Biol..

[37] WHO (1999). Management of Severe Malnutrition: A Manual for Physicians and Other Senior Health Workers.

[38] Bomers MK (2015). Rapid, accurate, and on-site detection of *C. difficile* in stool samples. Am. J. Gastroenterol..

