# Author Correction: Prediction of mortality in severe acute malnutrition in hospitalized children by faecal volatile organic compound analysis: proof of concept

**DOI:** 10.1038/s41598-021-83012-7

**Published:** 2021-02-03

**Authors:** Deborah A. van den Brink, Tim de Meij, Daniella Brals, Robert H. J. Bandsma, Johnstone Thitiri, Moses Ngari, Laura Mwalekwa, Nanne K. H. de Boer, Alfian Wicaksono, James A. Covington, Patrick F. van Rheenen, Wieger P. Voskuijl

**Affiliations:** 1 Department of Paediatrics, Centre for Liver, Digestive and Metabolic Diseases, University of Groningen, University Medical Centre Groningen, Groningen, The Netherlands; 2 Amsterdam Centre for Global Child Health, Emma Children’s Hospital, Amsterdam University Medical Centres, Amsterdam, The Netherlands; 3 Department of Global Health, Amsterdam Institute for Global Health and Development, Amsterdam University Medical Centres, Amsterdam, The Netherlands; 4 Department of Paediatric Gastroenterology, Emma, Children’s Hospital, Amsterdam University Medical Centres, Amsterdam, The Netherlands; 5 Department of Gastroenterology and Hepatology, Amsterdam Gastroenterology and Metabolism Research Institute, Amsterdam University Medical Centres, Amsterdam, The Netherlands; 6 Division of Gastroenterology, Hepatology and Nutrition and Translational Medicine Program, Hospital for Sick Children, Toronto, Canada; 7 The Childhood Acute Illness & Nutrition Network (CHAIN), Nairobi, Kenya; 8 KEMRI/Welcome Trust Research Programme, Kilifi, Kenya; 9 Department of Paediatrics, College of Medicine, University of Malawi, Blantyre, Malawi; 10 Department of Biomedical Sciences, College of Medicine, University of Malawi, Blantyre, Malawi; 11 School of Engineering, University of Warwick, Coventry, UK

Correction to: *Scientific Reports* 10.1038/s41598-020-75515-6, published online 05 November 2020

The Funding section in this Article is incomplete.

“The F75 study was funded by the Thrasher Research Fund, number 9403; analysis was supported by the CHAIN Network with funding from the Bill & Melinda Gates Foundation, grant number: OPP1131320; funders had no input in the design, data collection, analysis, or preparation of this manuscript. In addition, we would also like to acknowledge Owlstone FAIMS technology. We do want to clarify while we used the technology, at no point in time did they have any input in our data or analysis. There was also no funding or grants received from Owlstone.”

should read:

“The F75 study was funded by the Thrasher Research Fund, number 9403; analysis was supported by the CHAIN Network with funding from the Bill & Melinda Gates Foundation, grant number: OPP1131320, as well as Rotaryclub Purmerend Weidevenne; funders had no input in the design, data collection, analysis, or preparation of this manuscript. In addition, we would also like to acknowledge Owlstone FAIMS technology. We do want to clarify while we used the technology, at no point in time did they have any input in our data or analysis. There was also no funding or grants received from Owlstone.”

