# Supplementary material for: Prediction of mortality in severe acute malnutrition in hospitalized children by faecal volatile organic compound analysis: proof of concept
Source: Sci Rep. 2020 Nov 5;10:18785. doi: 10.1038/s41598-020-75515-6 (PMC7645771; doi:10.1038/s41598-020-75515-6)
Supplement: Supplementary file 1 — Supplementary Legends. [file 41598_2020_75515_MOESM1_ESM.docx]

**Supplementary Data:**

The following supplements are available:

Supplementary table S1: Complete classification results by Machine Learning (ML) algorithm, with best performance in bold.

Supplementary table S2: F75 trial daily record

Supplement 3: Raw FAIMS data in comparison groupings
