# Supplementary material for: Prediction of mortality in severe acute malnutrition in hospitalized children by faecal volatile organic compound analysis: proof of concept
Source: Sci Rep. 2020 Nov 5;10:18785. doi: 10.1038/s41598-020-75515-6 (PMC7645771; doi:10.1038/s41598-020-75515-6)
Supplement: Supplementary file 2 — Supplementary Information 1. [file 41598_2020_75515_MOESM2_ESM.docx]

**Appendix 1**

|  | Features | ML algorithm | Accuracy (95% CI) | Sensitivity (95% CI) | Specificity (95% CI) | PPV | NPV | P value |
| --- | --- | --- | --- | --- | --- | --- | --- | --- |
| Mortality vs. survival | 20 | SLR | 0.61 (0.44 - 0.77) | 0.95 (0.82 - 0.99) | 0.32 (0.13 - 0.57) | 0.73 | 0.75 | 0.10 |
|  |  | RF | 0.65 (0.50 - 0.80) | 0.63 (0.46 - 0.78) | 0.79 (0.54 - 0.94) | 0.86 | 0.52 | 0.03 |
|  |  | GP | 0.66 (0.52 - 0.80) | 0.55 (0.38 - 0.71) | 0.79 (0.54 - 0.94) | 0.84 | 0.47 | 0.03 |
|  |  | SVM | 0.66 (0.52 - 0.81) | 0.63 (0.46 - 0.78) | 0.79 (0.54 - 0.94) | 0.86 | 0.52 | 0.02 |
|  | 50 | SLR | 0.66 (0.51 - 0.81) | 0.55 (0.38 - 0.71) | 0.74 (0.49 - 0.91) | 0.81 | 0.45 | 0.02 |
|  |  | RF | 0.67 (0.52 - 0.82) | 0.61 (0.43 - 0.76) | 0.74 (0.49 - 0.91) | 0.82 | 0.48 | 0.02 |
|  |  | GP | 0.70 (0.56 - 0.85) | 0.71 (0.54 - 0.85) | 0.74 (0.49 - 0.91) | 0.84 | 0.56 | 0.01 |
|  |  | SVM | 0.65 (0.49 - 0.80) | 0.63 (0.46 - 0.78) | 0.79 (0.54 - 0.94) | 0.86 | 0.52 | 0.04 |
|  | 100 | SLR | 0.69 (0.54 - 0.84) | 0.97 (0.86 - 1.00) | 0.37 (0.16 - 0.62) | 0.76 | 0.88 | 0.01 |
|  |  | RF | 0.68 (0.53 - 0.83) | 0.79 (0.63 - 0.90) | 0.58 (0.33 - 0.80) | 0.79 | 0.58 | 0.01 |
|  |  | GP | 0.70 (0.54 - 0.86) | 0.76 (0.60 - 0.89) | 0.68 (0.43 - 0.87) | 0.83 | 0.59 | 0.01 |
|  |  | **SVM** | **0.71 (0.56 - 0.87)** | 0.76 (0.60 - 0.89) | 0.63 (0.38 - 0.84) | 0.81 | 0.57 | <0.01 |
| Early mortality vs. survival | 20 | SLR | 0.42 (0.19 - 0.65) | 0.11 (0.00 - 0.48) | 0.97 (0.86 - 1.00) | 0.50 | 0.82 | 0.23 |
|  |  | RF | 0.71 (0.52 - 0.90) | 0.89 (0.52 - 1.00) | 0.55 (0.38 - 0.71) | 0.32 | 0.95 | 0.03 |
|  |  | GP | 0.53 (0.29 - 0.78) | 0.33 (0.07 - 0.70) | 0.92 (0.79 - 0.98) | 0.50 | 0.85 | 0.39 |
|  |  | SVM | 0.50 (0.24 - 0.76) | 0.33 (0.07 - 0.70) | 0.92 (0.79 - 0.98) | 0.50 | 0.85 | 0.51 |
|  | 50 | SLR | 0.39 (0.17 - 0.60) | 0.56 (0.21 - 0.86) | 0.55 (0.38 - 0.71) | 0.23 | 0.84 | 0.15 |
|  |  | RF | 0.72 (0.54 - 0.91) | 0.67 (0.30 - 0.93) | 0.71 (0.54 - 0.85) | 0.35 | 0.90 | 0.02 |
|  |  | GP | 0.57 (0.33 - 0.81) | 0.44 (0.14 - 0.79) | 0.79 (0.63 - 0.90) | 0.33 | 0.86 | 0.26 |
|  |  | SVM | 0.49 (0.25 - 0.73) | 0.33 (0.07 - 0.70) | 0.82 (0.66 - 0.92) | 0.30 | 0.84 | 0.46 |
|  | 100 | SLR | 0.62 (0.40 - 0.85) | 0.44 (0.14 - 0.79) | 0.84 (0.69 - 0.94) | 0.40 | 0.86 | 0.13 |
|  |  | **RF** | **0.73 (0.57 - 0.90)** | 0.89 (0.52 - 1.00) | 0.55 (0.38 - 0.71) | 0.32 | 0.95 | 0.02 |
|  |  | GP | 0.54 (0.27 - 0.81) | 0.44 (0.14 - 0.79) | 0.84 (0.69 - 0.94) | 0.40 | 0.86 | 0.66 |
|  |  | SVM | 0.54 (0.29 - 0.79) | 0.56 (0.21 - 0.86) | 0.74 (0.57 - 0.87) | 0.33 | 0.88 | 0.35 |
| Late mortality vs. survival | 20 | SLR | 0.63 (0.39 - 0.87) | 0.74 (0.57 - 0.87) | 0.60 (0.26 - 0.88) | 0.88 | 0.38 | 0.11 |
|  |  | RF | 0.58 (0.36 - 0.80) | 0.95 (0.82 - 0.99) | 0.30 (0.07 - 0.65) | 0.84 | 0.60 | 0.23 |
|  |  | GP | 0.64 (0.42 - 0.86) | 0.74 (0.57 - 0.87) | 0.60 (0.26 - 0.88) | 0.88 | 0.38 | 0.09 |
|  |  | SVM | 0.59 (0.40 - 0.79) | 0.53 (0.36 - 0.69) | 0.80 (0.44 - 0.97) | 0.91 | 0.31 | 0.19 |
|  | 50 | **SLR** | **0.82 (0.67 - 0.96)** | 0.82 (0.66 - 0.92) | 0.70 (0.35 - 0.93) | 0.91 | 0.50 | <0.01 |
|  |  | RF | 0.59 (0.37 - 0.81) | 0.92 (0.79 - 0.98) | 0.30 (0.07 - 0.65) | 0.83 | 0.50 | 0.20 |
|  |  | GP | 0.63 (0.40 - 0.85) | 0.92 (0.79 - 0.98) | 0.40 (0.12 - 0.74) | 0.85 | 0.57 | 0.12 |
|  |  | SVM | 0.63 (0.43 - 0.84) | 0.79 (0.63 - 0.90) | 0.60 (0.26 - 0.88) | 0.88 | 0.43 | 0.10 |
|  | 100 | SLR | 0.79 (0.59 - 0.98) | 0.71 (0.54 - 0.85) | 0.90 (0.55 - 1.00) | 0.96 | 0.45 | <0.01 |
|  |  | RF | 0.60 (0.38 - 0.81) | 1.00 (0.91 - 1.00) | 0.20 (0.03 - 0.56) | 0.83 | 1.00 | 0.18 |
|  |  | GP | 0.44 (0.25 - 0.64) | 0.50 (0.33 - 0.67) | 0.60 (0.26 - 0.88) | 0.83 | 0.24 | 0.30 |
|  |  | SVM | 0.59 (0.39 - 0.79) | 0.55 (0.38 - 0.71) | 0.80 (0.44 - 0.97) | 0.91 | 0.32 | 0.20 |
| Early vs. late mortality | 20 | SLR | 0.54 (0.27 - 0.82) | 0.67 (0.30 - 0.93) | 0.60 (0.26 - 0.88) | 0.60 | 0.67 | 0.36 |
|  |  | RF | 0.71 (0.47 - 0.96) | 0.89 (0.52 - 1.00) | 0.50 (0.19 - 0.81) | 0.62 | 0.83 | 0.06 |
|  |  | GP | 0.66 (0.40 - 0.92) | 0.44 (0.14 - 0.79) | 0.90 (0.55 - 1.00) | 0.80 | 0.64 | 0.12 |
|  |  | SVM | 0.78 (0.54 - 1.00) | 0.89 (0.52 - 1.00) | 0.70 (0.35 - 0.93) | 0.73 | 0.88 | 0.02 |
|  | 50 | SLR | 0.62 (0.35 - 0.89) | 0.78 (0.40 - 0.97) | 0.50 (0.19 - 0.81) | 0.58 | 0.71 | 0.18 |
|  |  | RF | 0.72 (0.46 - 0.99) | 0.78 (0.40 - 0.97) | 0.80 (0.44 - 0.97) | 0.78 | 0.80 | 0.05 |
|  |  | GP | 0.50 (0.22 - 0.78) | 0.11 (0.00 - 0.48) | 1.00 (0.69 - 1.00) | 1.00 | 0.56 | 0.48 |
|  |  | **SVM** | **0.80 (0.57 - 1.00)** | 0.78 (0.40 - 0.97) | 0.80 (0.44 - 0.97) | 0.78 | 0.80 | 0.00 |
|  | 100 | SLR | 0.66 (0.40 - 0.92) | 0.56 (0.21 - 0.86) | 0.80 (0.44 - 0.97) | 0.71 | 0.67 | 0.11 |
|  |  | RF | 0.78 (0.55 - 1.00) | 0.78 (0.40 - 0.97) | 0.80 (0.44 - 0.97) | 0.78 | 0.80 | 0.02 |
|  |  | GP | 0.62 (0.36 - 0.89) | 0.56 (0.21 - 0.86) | 0.70 (0.35 - 0.93) | 0.63 | 0.64 | 0.18 |
|  |  | SVM | 0.68 (0.40 - 0.95) | 0.67 (0.30 - 0.93) | 0.80 (0.44 - 0.97) | 0.75 | 0.73 | 0.09 |
| SAM vs. no SAM | 20 | **SLR** | **0.99 (0.97 - 1.00)** | 0.98 (0.91 - 1.00) | 1.00 (0.59 - 1.00) | 1.00 | 0.88 | <0.01 |
|  |  | RF | 0.98 (0.95 - 1.00) | 0.96 (0.88 - 1.00) | 1.00 (0.59 - 1.00) | 1.00 | 0.78 | <0.01 |
|  |  | GP | 0.97 (0.93 - 1.00) | 0.96 (0.88 - 1.00) | 1.00 (0.59 - 1.00) | 1.00 | 0.78 | <0.01 |
|  |  | SVM | 0.96 (0.92 - 1.00) | 0.96 (0.88 - 1.00) | 1.00 (0.59 - 1.00) | 1.00 | 0.78 | <0.01 |
|  | 50 | **SLR** | **0.99 (0.98 - 1.00)** | 0.98 (0.91 - 1.00) | 1.00 (0.59 - 1.00) | 1.00 | 0.88 | <0.01 |
|  |  | RF | 0.97 (0.94 - 1.00) | 0.96 (0.88 - 1.00) | 1.00 (0.59 - 1.00) | 1.00 | 0.78 | <0.01 |
|  |  | GP | 0.97 (0.93 - 1.00) | 0.95 (0.85 - 0.99) | 1.00 (0.59 - 1.00) | 1.00 | 0.70 | <0.01 |
|  |  | SVM | 0.98 (0.95 - 1.00) | 0.96 (0.88 - 1.00) | 1.00 (0.59 - 1.00) | 1.00 | 0.78 | <0.01 |
|  | 100 | **SLR** | **0.99 (0.98 - 1.00)** | 0.96 (0.88 - 1.00) | 1.00 (0.59 - 1.00) | 1.00 | 0.78 | <0.01 |
|  |  | RF | 0.98 (0.95 - 1.00) | 0.96 (0.88 - 1.00) | 1.00 (0.59 - 1.00) | 1.00 | 0.78 | <0.01 |
|  |  | GP | 0.98 (0.96 - 1.00) | 0.98 (0.91 - 1.00) | 1.00 (0.59 - 1.00) | 1.00 | 0.88 | <0.01 |
|  |  | SVM | 0.97 (0.93 - 1.00) | 0.89 (0.78 - 0.96) | 1.00 (0.59 - 1.00) | 1.00 | 0.54 | <0.01 |
| WAZ ≤ -3 vs. WAZ > -3 | 20 | **SLR** | **0.70 (0.54 - 0.85)** | 0.91 (0.59 - 1.00) | 0.46 (0.31 - 0.61) | 0.29 | 0.95 | 0.02 |
|  |  | RF | 0.60 (0.44 - 0.76) | 1.00 (0.72 - 1.00) | 0.35 (0.21 - 0.50) | 0.27 | 1.00 | 0.15 |
|  |  | GP | 0.44 (0.22 - 0.65) | 0.45 (0.17 - 0.77) | 0.63 (0.48 - 0.77) | 0.23 | 0.83 | 0.26 |
|  |  | SVM | 0.59 (0.42 - 0.76) | 1.00 (0.72 - 1.00) | 0.24 (0.13 - 0.39) | 0.24 | 1.00 | 0.18 |
|  | 50 | SLR | 0.69 (0.53 - 0.85) | 0.82 (0.48 - 0.98) | 0.57 (0.41 - 0.71) | 0.31 | 0.93 | 0.02 |
|  |  | RF | 0.57 (0.40 - 0.74) | 0.73 (0.39 - 0.94) | 0.52 (0.37 - 0.67) | 0.27 | 0.89 | 0.23 |
|  |  | GP | 0.55 (0.32 - 0.77) | 0.55 (0.23 - 0.83) | 0.70 (0.54 - 0.82) | 0.30 | 0.86 | 0.67 |
|  |  | SVM | 0.56 (0.37 - 0.75) | 0.82 (0.48 - 0.98) | 0.39 (0.25 - 0.55) | 0.24 | 0.90 | 0.26 |
|  | 100 | **SLR** | **0.70 (0.54 - 0.86)** | 0.73 (0.39 - 0.94) | 0.70 (0.54 - 0.82) | 0.36 | 0.91 | 0.02 |
|  |  | RF | 0.56 (0.39 - 0.74) | 0.82 (0.48 - 0.98) | 0.48 (0.33 - 0.63) | 0.27 | 0.92 | 0.25 |
|  |  | GP | 0.62 (0.39 - 0.85) | 0.73 (0.39 - 0.94) | 0.70 (0.54 - 0.82) | 0.36 | 0.91 | 0.11 |
|  |  | SVM | 0.53 (0.33 - 0.73) | 0.64 (0.31 - 0.89) | 0.52 (0.37 - 0.67) | 0.24 | 0.86 | 0.63 |
| Age > 2 yrs vs. age ≤ 2 yrs | 20 | SLR | 0.66 (0.51 - 0.82) | 0.60 (0.36 - 0.81) | 0.70 (0.53 - 0.84) | 0.52 | 0.76 | 0.02 |
|  |  | **RF** | **0.79 (0.66 - 0.92)** | 0.75 (0.51 - 0.91) | 0.76 (0.59 - 0.88) | 0.63 | 0.85 | <0.01 |
|  |  | GP | 0.66 (0.51 - 0.81) | 0.80 (0.56 - 0.94) | 0.49 (0.32 - 0.66) | 0.46 | 0.82 | 0.03 |
|  |  | SVM | 0.69 (0.55 - 0.84) | 0.70 (0.46 - 0.88) | 0.65 (0.47 - 0.80) | 0.52 | 0.80 | 0.01 |
|  | 50 | SLR | 0.53 (0.36 - 0.69) | 0.65 (0.41 - 0.85) | 0.49 (0.32 - 0.66) | 0.41 | 0.72 | 0.36 |
|  |  | RF | 0.68 (0.53 - 0.83) | 0.55 (0.32 - 0.77) | 0.81 (0.65 - 0.92) | 0.61 | 0.77 | 0.01 |
|  |  | GP | 0.45 (0.30 - 0.60) | 0.60 (0.36 - 0.81) | 0.54 (0.37 - 0.71) | 0.41 | 0.71 | 0.27 |
|  |  | SVM | 0.62 (0.47 - 0.78) | 0.80 (0.56 - 0.94) | 0.49 (0.32 - 0.66) | 0.46 | 0.82 | 0.06 |
|  | 100 | SLR | 0.50 (0.34 - 0.67) | 0.65 (0.41 - 0.85) | 0.51 (0.34 - 0.68) | 0.42 | 0.73 | 0.48 |
|  |  | RF | 0.66 (0.51 - 0.82) | 0.80 (0.56 - 0.94) | 0.51 (0.34 - 0.68) | 0.47 | 0.83 | 0.02 |
|  |  | GP | 0.43 (0.28 - 0.59) | 0.70 (0.46 - 0.88) | 0.38 (0.22 - 0.55) | 0.38 | 0.70 | 0.21 |
|  |  | SVM | 0.60 (0.44 - 0.76) | 0.85 (0.62 - 0.97) | 0.41 (0.25 - 0.58) | 0.44 | 0.83 | 0.11 |
| Oedema vs. no oedema | 20 | **SLR** | **0.71 (0.56 - 0.87)** | 0.77 (0.55 - 0.92) | 0.66 (0.48 - 0.81) | 0.59 | 0.82 | <0.01 |
|  |  | RF | 0.61 (0.45 - 0.76) | 0.36 (0.17 - 0.59) | 0.91 (0.77 - 0.98) | 0.73 | 0.70 | 0.09 |
|  |  | GP | 0.68 (0.53 - 0.82) | 0.36 (0.17 - 0.59) | 0.94 (0.81 - 0.99) | 0.80 | 0.70 | 0.01 |
|  |  | SVM | 0.59 (0.41 - 0.77) | 0.41 (0.21 - 0.64) | 0.94 (0.81 - 0.99) | 0.82 | 0.72 | 0.14 |
|  | 50 | **SLR** | **0.71 (0.56 - 0.86)** | 0.77 (0.55 - 0.92) | 0.69 (0.51 - 0.83) | 0.61 | 0.83 | <0.01 |
|  |  | RF | 0.62 (0.47 - 0.78) | 0.36 (0.17 - 0.59) | 0.94 (0.81 - 0.99) | 0.80 | 0.70 | 0.06 |
|  |  | GP | 0.66 (0.51 - 0.82) | 0.59 (0.36 - 0.79) | 0.71 (0.54 - 0.85) | 0.57 | 0.74 | 0.02 |
|  |  | SVM | 0.52 (0.34 - 0.69) | 0.50 (0.28 - 0.72) | 0.77 (0.60 - 0.90) | 0.58 | 0.71 | 0.41 |
|  | 100 | SLR | 0.64 (0.48 - 0.80) | 0.45 (0.24 - 0.68) | 0.89 (0.73 - 0.97) | 0.71 | 0.72 | 0.04 |
|  |  | RF | 0.62 (0.46 - 0.78) | 0.36 (0.17 - 0.59) | 0.94 (0.81 - 0.99) | 0.80 | 0.70 | 0.07 |
|  |  | **GP** | **0.71 (0.56 - 0.85)** | 0.68 (0.45 - 0.86) | 0.69 (0.51 - 0.83) | 0.58 | 0.77 | <0.01 |
|  |  | SVM | 0.52 (0.35 - 0.69) | 0.45 (0.24 - 0.68) | 0.77 (0.60 - 0.90) | 0.56 | 0.69 | 0.41 |
| Diarrhoea vs. no diarrhoea | 20 | SLR | 0.55 (0.38 - 0.71) | 0.64 (0.41 - 0.83) | 0.54 (0.37 - 0.71) | 0.47 | 0.70 | 0.28 |
|  |  | RF | 0.46 (0.30 - 0.62) | 0.55 (0.32 - 0.76) | 0.63 (0.45 - 0.79) | 0.48 | 0.69 | 0.31 |
|  |  | GP | 0.57 (0.40 - 0.73) | 0.41 (0.21 - 0.64) | 0.83 (0.66 - 0.93) | 0.60 | 0.69 | 0.21 |
|  |  | SVM | 0.62 (0.46 - 0.78) | 0.50 (0.28 - 0.72) | 0.83 (0.66 - 0.93) | 0.65 | 0.73 | 0.06 |
|  | 50 | SLR | 0.58 (0.42 - 0.74) | 0.50 (0.28 - 0.72) | 0.77 (0.60 - 0.90) | 0.58 | 0.71 | 0.16 |
|  |  | RF | 0.58 (0.42 - 0.74) | 0.55 (0.32 - 0.76) | 0.71 (0.54 - 0.85) | 0.55 | 0.71 | 0.16 |
|  |  | GP | 0.62 (0.47 - 0.78) | 0.45 (0.24 - 0.68) | 0.83 (0.66 - 0.93) | 0.63 | 0.71 | 0.06 |
|  |  | SVM | 0.64 (0.48 - 0.80) | 0.50 (0.28 - 0.72) | 0.86 (0.70 - 0.95) | 0.69 | 0.73 | 0.04 |
|  | 100 | SLR | 0.59 (0.42 - 0.75) | 0.55 (0.32 - 0.76) | 0.77 (0.60 - 0.90) | 0.60 | 0.73 | 0.13 |
|  |  | RF | 0.57 (0.41 - 0.73) | 0.59 (0.36 - 0.79) | 0.63 (0.45 - 0.79) | 0.50 | 0.71 | 0.18 |
|  |  | GP | 0.63 (0.48 - 0.79) | 0.36 (0.17 - 0.59) | 0.89 (0.73 - 0.97) | 0.67 | 0.69 | 0.05 |
|  |  | **SVM** | **0.66 (0.51 - 0.81)** | 0.45 (0.24 - 0.68) | 0.89 (0.73 - 0.97) | 0.71 | 0.72 | 0.02 |
| Pneumonia vs. no pneumonia | 20 | SLR | 0.58 (0.41 - 0.74) | 0.59 (0.33 - 0.82) | 0.60 (0.43 - 0.75) | 0.38 | 0.77 | 0.81 |
|  |  | RF | 0.55 (0.37 - 0.74) | 0.35 (0.14 - 0.62) | 0.93 (0.80 - 0.98) | 0.67 | 0.77 | 0.74 |
|  |  | GP | 0.59 (0.41 - 0.76) | 0.59 (0.33 - 0.82) | 0.68 (0.51 - 0.81) | 0.43 | 0.79 | 0.16 |
|  |  | SVM | 0.56 (0.37 - 0.75) | 0.47 (0.23 - 0.72) | 0.78 (0.62 - 0.89) | 0.47 | 0.78 | 0.76 |
|  | 50 | SLR | 0.57 (0.40 - 0.74) | 0.53 (0.28 - 0.77) | 0.68 (0.51 - 0.81) | 0.41 | 0.77 | 0.79 |
|  |  | RF | 0.53 (0.34 - 0.71) | 0.59 (0.33 - 0.82) | 0.60 (0.43 - 0.75) | 0.38 | 0.77 | 0.38 |
|  |  | GP | 0.62 (0.45 - 0.79) | 0.65 (0.38 - 0.86) | 0.68 (0.51 - 0.81) | 0.46 | 0.82 | 0.08 |
|  |  | SVM | 0.53 (0.36 - 0.70) | 0.18 (0.04 - 0.43) | 1.00 (0.91 - 1.00) | 1.00 | 0.74 | 0.37 |
|  | 100 | SLR | 0.53 (0.35 - 0.71) | 0.41 (0.18 - 0.67) | 0.75 (0.59 - 0.87) | 0.41 | 0.75 | 0.66 |
|  |  | RF | 0.52 (0.33 - 0.70) | 0.53 (0.28 - 0.77) | 0.73 (0.56 - 0.85) | 0.45 | 0.78 | 0.43 |
|  |  | **GP** | **0.63 (0.47 - 0.80)** | 0.59 (0.33 - 0.82) | 0.73 (0.56 - 0.85) | 0.48 | 0.81 | 0.06 |
|  |  | SVM | 0.55 (0.38 - 0.71) | 0.76 (0.50 - 0.93) | 0.38 (0.23 - 0.54) | 0.34 | 0.79 | 0.30 |

**Table S1: Complete classification results by Machine Learning (ML) algorithm, with best performance in bold.**

Definitions: SAM, severe acute malnutrition; HIV, human immunodeficiency virus 1; WAZ, weight for age Z-score; SLR, Sparse Logistic Regression; RF, Random Forrest; GP, Gaussian Process; and SVM, Support Vector Machine.

**Appendix 2**

| Month __ __ Date | __ __ |
| --- | --- |
| Weight *kg* now | __ __ · __ __ |
| MUAC *cm* now | __ __ · __ |
| Oedema now | +++ ++ + N |
| **Clinical Features** | |
| Lower chest wall indrawing in 24h | Y N |
| Had oxygen or SaO2<90% in last 24h | Y N |
| *Shock in last 24h | Y N |
| Congestive cardiac failure in last 24 h | Y N |
| Lowest AVPU in last 24h | A V P U |
| Convulsions in last 24h | Y N |
| Hypoglycaemia <3 mmol/l in last 24h | Y N |
| Vomiting in last 24h | Y N |
| Diarrhoea (3+ loose/watery) in 24h | Y N |
| Temperature 38.5^o^C or more in 24h | Y N |
| Temperature less than 36.5^o^C in 24h | Y N |
| **Any WHO dangers signs now | Y N |
| **Medical & Nutritional Treatment** | |
| **S**tabilization, **T**ransition, **R**ehab | S T R |
| NG tube in last 24h | Y N |
| Any EBM or breastfeeding in 24h | Y N |
| Currently on F75 | Y N |
| Currently on F100 | Y N |
| Currently on RUTF | Y N |
| Non-standard formula milk e.g. soya | Y N |
| Observed to complete feeds | Y N |
| ReSoMal in last 24h | Y N |
| IV fluids given in last 24h | Y N |
| Blood transfusion in last 24h | Y N |
| Currently on IV antibiotics | 1^st^ 2^nd^ No |
| Anti-TB treatment currently | Y N |
| **Labs** | |
| Haemoglobin <5g/dl | Y N |

**Table S2: F75 trial daily record**
